# Validação do modelo lógico da assistência integral às crianças com síndrome congênita da Zika

**DOI:** 10.1590/0102-311XPT051924

**Published:** 2025-07-04

**Authors:** Danielle Amaral de Freitas, Reinaldo Souza-Santos, Sónia Dias, Rosa Maria Soares Madeira Domingues, Mayumi Duarte Wakimoto

**Affiliations:** 1 Escola Nacional de Saúde Pública Sergio Arouca, Fundação Oswaldo Cruz, Rio de Janeiro, Brasil.; 2 Universidade Federal do Rio de Janeiro, Rio de Janeiro, Brasil.; 3 Universidade NOVA de Lisboa, Lisboa, Portugal.; 4 Instituto Nacional de Infectologia Evandro Chagas, Fundação Oswaldo Cruz, Rio de Janeiro, Brasil.

**Keywords:** Infecção por Zika Vírus, Avaliação em Saúde, Saúde Materno-Infantil, Modelos de Assistência à Saúde, Assistência Integral à Saúde, Zika Virus Infection, Health Evaluation, Maternal and Child Health, Healthcare Models, Comprehensive Health Care, Infección por el Virus Zika, Evaluación en Salud, Salud Materno-Infantil, Modelos de Atención de Salud, Atención Integral de Salud

## Abstract

O objetivo deste estudo foi elaborar e validar o modelo lógico dos componentes da assistência integral às crianças com síndrome congênita da Zika (SCZ) no Município do Rio de Janeiro, Brasil. Foram desenvolvidas três etapas: (1) busca na literatura científica e em documentos oficiais para elaboração do modelo lógico; (2) aperfeiçoamento do modelo incorporando as sugestões de especialistas nas áreas de saúde da criança, da pessoa com deficiência, avaliação em saúde e Rede de Atenção à Saúde (RAS); e (3) validação do modelo lógico por meio do método de consenso de Delphi em duas fases: avaliação da pertinência e da relevância dos itens do modelo através do percentual de concordância (PC), do coeficiente alfa de Cronbach, mediana e valor interquartil. O modelo lógico contém todos os componentes necessários para o acolhimento e acompanhamento dos casos de SCZ, desde a concepção até o terceiro ano de vida. Foram classificados como pertinentes 136 itens do modelo (fase 1: PC 96-97% e 0,76-0,93; fase 2: PC 98% e 0,88-0,97), 98% muito relevantes e 2% relevantes. A elaboração e validação do modelo lógico possibilitou a representação gráfica dos componentes necessários para assistência integral à saúde das crianças com SCZ, bem como a organização dos fluxos assistenciais, com os elementos necessários para auxiliar a gestão local no planejamento, estruturação e avaliação da RAS. Apesar das características singulares da cidade, este modelo revela-se passível de aplicação em outras localidades com fatores contextuais semelhantes aos observados no Rio de Janeiro.

## Abstract (en)

This study aimed to develop and validate a logical model of the components of comprehensive care for children with congenital Zika syndrome (CZS) in the municipality of Rio de Janeiro, Brazil, in three stages: (1) search in the scientific literature and official documents to elaborate the logical model, (2) improvement of the model by incorporating the suggestions of specialists in child health, people with disabilities, health evaluation, and health care network, and (3) validation of the logical model by the Delphi consensus in two phases: evaluation of the relevance of the model items by the percentage of agreement (PA), Cronbach’s alpha coefficient, medians, and interquartile values. The logical model contains all the necessary components to admit and follow up CZS cases from conception to the third year of life. A total of 136 items of the model were classified as relevant (phase 1: PA 96-97% and 0.76-0.93; phase 2: PA 98% and 0.88-0.97), 98% very relevant and 2% relevant. The elaboration and validation of the logical model could graphically represent the components necessary for comprehensive health care for children with CZS and organize care flows with the necessary elements to assist local management in planning, structuring, and evaluating the health care network. Despite the unique characteristics of the municipality, this model may serve other locations with contextual factors resembling those in Rio de Janeiro.

## Resumen

El objetivo de este estudio fue desarrollar y validar el modelo lógico de los componentes de la atención integral para niños con síndrome congénito del Zika (SCZ) en la ciudad de Rio de Janeiro, Brasil. Se desarrollaron tres etapas: (1) búsqueda en la literatura científica y en documentos oficiales para elaborar el modelo lógico; (2) mejora del modelo con la inclusión de las sugerencias de especialistas en las áreas de salud infantil, personas con discapacidad, evaluación de la salud y red asistencial; y (3) validación del modelo lógico mediante el método de consenso Delphi en dos fases: evaluación de la relevancia y pertinencia de los ítems del modelo mediante el porcentaje de concordancia (PC), el coeficiente alfa de Cronbach, la mediana y el valor intercuartílico. El modelo lógico contiene todos los componentes necesarios para la recepción y seguimiento de los casos de SCZ, desde la concepción hasta el tercer año de vida. Se clasificaron 136 ítems del modelo como relevantes (fase 1: PC 96-97% y 0,76-0,93; fase 2: PC 98% y 0,88-0,97), 98% muy relevante y 2% relevante. La elaboración y validación del modelo lógico permitió la representación gráfica de los componentes necesarios para la atención integral en salud de niños con SCZ, así como la organización de flujos de atención, con los elementos necesarios para asistir a la gestión local en la planificación, estructuración y evaluación de la red de atención en salud. A pesar de las características únicas de la ciudad, este modelo demuestra ser aplicable en otras ubicaciones con factores contextuales similares a los observados en Rio de Janeiro.

## Introdução

A síndrome congênita da Zika (SCZ) ^1^ possui sinais e sintomas semelhantes aos encontrados em outras infecções congênitas, descritas pelo acrônimo STORCH (sífilis, toxoplasmose, rubéola, citomegalovírus, herpes simples) ^2^. Elas estão associadas a danos físicos e mentais, quadro que demanda o cuidado integral e especializado com suporte assistencial e social ^3^.

No Brasil, entre 2015 e 2020, foram notificados 19.622 casos suspeitos de SCZ e 3.563 confirmados. Entre as crianças com SCZ, apenas 52% (1.847) foram acompanhadas na atenção primária à saúde (APS) e 49% (1.746), em atendimento especializado, sugerindo a existência de barreiras de acesso a serviços de saúde ^4^. Diversos fatores podem constituir entraves para o adequado cuidado e acompanhamento das crianças com SCZ, muitos deles comuns àqueles vivenciados por pessoas com deficiência e necessidades especiais, tais como: a demora no diagnóstico; a necessidade de diferentes especialistas; a dependência do paciente em um contexto familiar de vulnerabilidade social; a comunicação insuficiente entre os serviços especializados, resultando em assistência fragmentada; ausência de estruturação de uma rede de referência e contrarreferência; falhas no sistema de regulação, ocasionando alto tempo de espera para consultas e exames; assistência em locais distantes da residência, com percursos longos para unidades de saúde, gerando maior custo e gasto de tempo; desconhecimento do fluxo de atendimento nos serviços especializados; e equívocos no processo de encaminhamento pelos profissionais de saúde ^5^ ^,^ ^6^ ^,^ ^7^ ^,^ ^8^ ^,^ ^9^ ^,^ ^10^ ^,^ ^11^.

A assistência adequada às crianças com SCZ envolve desde procedimentos com menor densidade tecnológica na APS até o atendimento em hospitais especializados, o que pressupõe a organização da Rede de Atenção à Saúde (RAS) como capaz de fornecer cuidado integral ^12^. Portanto, é essencial conhecer e avaliar o plano de cuidados às crianças com SCZ ^13^, assim como os fluxos assistenciais definidos entre os níveis de atenção ^14^ ^,^ ^15^.

A avaliação do cuidado às crianças com SCZ pode ser feita por meio de um modelo lógico, que representa visualmente o funcionamento do programa, suas causas e resultados esperados. Esse esquema integra o raciocínio avaliativo ao design estrutural, mostrando a relação entre causas imediatas, distais e os objetivos da intervenção ^16^ ^,^ ^17^ ^,^ ^18^ ^,^ ^19^ ^,^ ^20^ ^,^ ^21^ ^,^ ^22^. Ele pode permitir aos gestores e avaliadores uma visão mais clara sobre a racionalidade da construção do tratamento ^23^.

Estudos avaliativos anteriores evidenciaram a necessidade de uma ferramenta para avaliar a RAS integrando diferentes áreas do conhecimento. Pesquisas apontaram deficiências na estrutura e nas ações da atenção pré-natal, na Rede de Cuidados à Pessoa com Deficiência, na resposta à emergência da microcefalia associada ao Zika vírus (ZIKV) e na APS, destacando falhas na coordenação, integralidade e monitoramento dos serviços e orientação familiar e comunitária ^24^ ^,^ ^25^ ^,^ ^26^ ^,^ ^27^. Portanto, na ausência de um modelo lógico que possa ser base para futuros estudos avaliativos da RAS em relação às crianças com SCZ e que possibilite a sistematização de uma rede de cuidados com longitudinalidade e integralidade para casos tão complexos como os dessas crianças, este estudo tem por objetivo apresentar o desenvolvimento e validação do modelo lógico dos componentes da assistência integral às crianças com SCZ na RAS da cidade do Rio de Janeiro, Brasil.

## Métodos

Trata-se de um estudo de desenvolvimento e validação do modelo lógico da RAS de crianças com SCZ. O modelo lógico foi desenvolvido e validado em três etapas entre 2019 e 2020: (1) definição do problema e do contexto e elaboração do modelo lógico; (2) aperfeiçoamento do modelo lógico; e (3) validação do modelo lógico ^16^ ^,^ ^18^ ^,^ ^28^ ^,^ ^29^ ^,^ ^30^ ^,^ ^31^.

### Primeira etapa: definição do problema e do contexto e elaboração do modelo lógico

Inicialmente, foi realizada busca por documentos oficiais nos sites do Ministério da Saúde brasileiro e da Organização Mundial da Saúde (OMS) sobre Zika, Zika em gestantes e SCZ, além de políticas sobre atenção integral à saúde da mulher, da criança e da pessoa com deficiência. Também foram procuradas referências bibliográficas sobre protocolos e diretrizes para o manejo clínico na Biblioteca Virtual em Saúde utilizando a chave de busca: (tw:(Zika)) AND ((tw:(protocolo*)) OR (tw:(diretriz*))) AND ((tw:(criança*)) OR (tw:(Gesta*))). O recorte temporal foi entre 2014 e fevereiro de 2020. Foram encontrados 260 documentos; destes, 12 foram selecionados para análise qualitativa, e os demais foram excluídos por não tratarem de gestantes com infecção suspeita/confirmada pelo ZIKV ou crianças com SCZ ^32^ ^,^ ^33^ ^,^ ^34^ ^,^ ^35^ ^,^ ^36^ ^,^ ^37^ ^,^ ^38^.

A partir dessas referências normativas, foi elaborado um modelo lógico inicial, incluindo os componentes e subcomponentes da RAS necessários para o cuidado integral às crianças com SCZ, do nascimento até o 3º ano de vida. A faixa etária escolhida foi baseada nos protocolos nacionais ^39^.

### Segunda etapa: aperfeiçoamento do modelo lógico

Foram convidados especialistas considerando pelo menos um dos seguintes critérios: (1) atuação nas áreas de ensino, pesquisa e/ou extensão; (2) conhecimento sobre os temas: “Rede de Atenção à Saúde”, “cuidado integral à saúde das crianças” e/ou “cuidado integral à pessoa com deficiência”; (3) especialista da área de avaliação de serviços/programas de saúde.

Foram realizadas duas rodadas para sugestões e ajustes, feitas por e-mail e por videoconferência individualmente com cada especialista. O objetivo foi avaliar a abordagem teórica e normativa da assistência e do fluxo assistencial às crianças com SCZ à luz do conhecimento atual e de acordo com a prática nos serviços de saúde.

Na primeira rodada, foi enviado o modelo lógico com questionário contendo perguntas abertas para verificar a conformidade. Na segunda, após a inclusão das alterações sugeridas, foi enviado modelo para aprovação e prosseguimento para a etapa seguinte.

### Terceira etapa: validação do modelo lógico

Foi utilizado o método Delphi, por meio da técnica de consenso, para validação do modelo lógico, visando garantir a sua validade de conteúdo. O método tem como princípios: iteração, *feedback* controlado, resposta estatística do grupo, interação estruturada e anonimato ou “quase anonimato” ^40^.

#### Amostra e seleção dos participantes

Foi utilizada amostra de conveniência, composta por 20 especialistas (pesquisadores, gestores ou profissionais de saúde) com reconhecida experiência nas áreas de atenção à saúde da criança, das pessoas com deficiência e da avaliação de sistemas, programas e serviços de saúde. Eles foram selecionados por indicação de integrantes do grupo técnico que atuam no cuidado às crianças com síndromes congênitas no Estado do Rio de Janeiro.

A validação do modelo incluiu a avaliação, em dois momentos distintos, da pertinência e da relevância dos itens, tendo sido elaborado um questionário específico para cada momento (questionários 1 e 2). Foi enviado e-mail convite para os 20 especialistas contendo o Termo de Consentimento Livre e Esclarecido, o modelo lógico a ser avaliado, as orientações do preenchimento, o resumo sobre avaliação do modelo e o questionário 1 para a avaliação da pertinência. Ele foi dividido em cinco partes: (1) identificação do participante, (2) avaliação da estrutura, (3) processo, (4) produto, e (5) resultado a curto e médio prazo e impacto; com questões dicotômicas (“É pertinente?” e “Não é pertinente?”) para cada item e uma pergunta aberta para o registro de recomendações em cada componente.

Após análise das respostas e inclusão dos ajustes, foi encaminhado o questionário 2 para os profissionais que haviam respondido o primeiro, visando à avaliação da relevância dos itens do modelo. Cada item poderia ser avaliado como “muito relevante”, “relevante” e “pouco relevante”, com atribuição dos seguintes pesos: muito relevante = 3, relevante = 2 e pouco relevante = 1 ^41^ ^,^ ^42^.

O prazo para o envio da resposta foi de 15 dias a partir da data do envio de cada questionário. Para aumentar a taxa de resposta, foram enviados e-mails antes do prazo, na data de expiração e outros três e-mails após o prazo estabelecido quando necessário.

#### Análise de dados

Para análise do consenso na avaliação da pertinência, foi utilizado o percentual de concordância simples por item, por elementos do modelo lógico (estrutura, atividade, produto e resultado) e do modelo completo. O percentual de concordância simples entre avaliadores foi medido com variação entre 0 e 1, sendo: sem concordância (entre 0 e 0,3); fraca (entre 0,31 e 0,5); moderada (entre 0,51 e 0,7); forte (entre 0,71 e 0,9); muito forte (entre 0,91 e 1) ^43^. Foram considerados concordantes os itens com valores classificados como fortes, ou seja, maiores que 70% ^42^ ^,^ ^44^ ^,^ ^45^.

Para análise do consenso na avaliação da relevância, foram utilizadas as seguintes métricas: a mediana, o intervalo interquartil e a concordância entre avaliadores. Com relação à mediana e ao intervalo interquartil, o valor de cada item do modelo foi estabelecido seguindo os critérios: quando o valor do intervalo interquartil foi igual a zero, o valor do item era igual à mediana; quando o valor do intervalo interquartil foi 0,5, o valor do item era igual ao valor com maior percentual de resposta; quando o valor do intervalo interquartil foi 0,75 e os valores apresentaram empate no percentual de resposta, o grupo de pesquisa definiu o nível de relevância com base na literatura ^43^ ^,^ ^46^ ^,^ ^47^.

Já para análise da consistência interna foi verificada a confiabilidade entre examinadores através do coeficiente alfa de Cronbach, cujos valores variam entre 0 e 1. Para valores entre 0 e 0,2, a consistência é pequena; entre 0,21 e 0,4, razoável; entre 0,41 a 0,6, moderada; entre 0,61 e 0,8, substancial; e entre 0,81 e 1, quase perfeita ^44^ ^,^ ^45^. Após consenso e valoração dos elementos, o modelo lógico final foi encaminhado para os participantes para aprovação. Foram utilizados os softwares Microsoft Excel 365 (https://products.office.com/) e SPSS 20.0 (https://www.ibm.com/).

O estudo foi aprovado pelo Comitê de Ética em Pesquisa da Escola Nacional de Saúde Pública Sergio Arouca, Fundação Oswaldo Cruz (CAAE: 94162218.5.0000.5240).

## Resultados

### Primeira etapa: definição do problema e do contexto e construção do modelo lógico

O modelo lógico inicial continha os elementos estrutura, processo e resultado, incluindo quatro componentes relacionados aos níveis de atenção à saúde no Brasil, oito subcomponentes, sete itens relativos à estrutura, 38 ao processo e 16 ao resultado (Material Suplementar - Figuras S1, S2, S3 e S4; https://cadernos.ensp.fiocruz.br/static//arquivo/suppl-e00051924_4462.pdf).

### Segunda etapa: aperfeiçoamento do modelo lógico

Participaram desta etapa três especialistas, todas atuando na área de pesquisa, com experiência na RAS: uma na área de conhecimento materno-infantil; uma na área materno-infantil e na avaliação de serviços/programas de saúde; e uma na área de saúde integral à pessoa com deficiência.

Quanto à estrutura, o modelo foi adaptado ao proposto pelo Centro de Controle e Prevenção de Doenças dos Estados Unidos ^22^, contendo os elementos: estrutura, atividades/processo, produto, resultado (curto e médio prazo) e impacto, incluindo os aspectos contextuais segundo modelo tipo 2, descrito por Mills et al. ^48^. Foram adicionados ao modelo dois componentes e dois subcomponentes. Quanto aos itens, permaneceram três itens relativos à estrutura, adaptados a cada subcomponente, 37 relacionados ao processo, 34 ao produto, 31 aos resultados (curto 29, médio prazo 1 e relativo ao impacto 1).

### Terceira etapa: validação do modelo lógico

#### Avaliação da pertinência dos itens do modelo lógico

Dos 20 especialistas convidados, sete (35%) responderam aos questionários enviados. Todos os respondentes são profissionais da área da saúde, sendo cinco do sexo feminino. Todos apresentavam pós-graduação *stricto sensu* (um, pós-doutorado; três, doutorado; e três, mestrado), com atuação nas áreas de gestão (n = 3), pesquisa (n = 3) e assistência (n = 1). Um respondente apresentava até cinco anos de atuação; os demais, mais de 15 anos.

A maioria (86%) dos itens do modelo lógico teve 100% de concordância. O percentual de concordância do modelo lógico e dos componentes variou entre 95 e 98%. O coeficiente alfa de Cronbach foi considerado “quase perfeito” para o modelo completo e para os itens que compõem a estrutura e o produto. Para os itens que compõem o processo e o resultado, o coeficiente foi “substancial” (Material Suplementar - Tabela S1; https://cadernos.ensp.fiocruz.br/static//arquivo/suppl-e00051924_4462.pdf). Como o percentual de concordância e o coeficiente alfa de Cronbach demonstraram concordância congruente, foram mantidos todos os itens do modelo lógico.

#### Avaliação da relevância dos itens do modelo lógico

Dos sete especialistas que participaram da avaliação da pertinência, seis responderam ao questionário para avaliação da relevância. Entre os 136 itens do modelo lógico, 134 foram considerados “muito relevantes” e dois “relevantes”. O percentual de concordância simples teve resultado entre 83 e 100% em 112 itens, 67% em 19 itens e 50% em cinco itens. A mediana variou entre 2,5 e 3 e o intervalo interquartil variou entre 0 e 0,75. O nível de concordância entre os avaliadores foi considerado “muito forte” em 128 itens do modelo lógico e “forte” em oito itens. O alfa de Cronbach foi considerado “quase perfeito” para todo o modelo e para todos os componentes que o compõem (Material Suplementar - Tabela S1; https://cadernos.ensp.fiocruz.br/static//arquivo/suppl-e00051924_4462.pdf).

#### O modelo lógico dos componentes para assistência integral às crianças com SCZ

O modelo lógico final contém 136 itens, sendo 33 relativos à estrutura, 38 ao processo, 33 ao produto e 32 aos resultados (Quadros 1, 2, 3, 4 e 5). O componente “Atenção primária à saúde” (Quadros 1 e 2) contém 40% (54) dos itens que compõem o modelo lógico; o componente “Atenção secundária à saúde” (ASS) (Quadro 3), 18% (25); e o componente “Atenção hospitalar” (Quadro 4), 27% (37). Todos os itens da APS, da ASS e da Atenção hospitalar foram considerados “muito relevantes”.

O componente “Central municipal de regulação” (Quadro 5) constituiu 4% (6) dos itens do modelo, sendo um item considerado “relevante” e os demais “muito relevantes”. O componente “Vigilância em saúde” (Quadro 5) contribuiu com 11% (14) dos itens do modelo, sendo dois considerados “relevantes” e os demais “muito relevantes”.

**Quadro 1:** Modelo lógico validado dos componentes da assistência às crianças com síndrome congênita da Zika (SCZ) na atenção primária à saúde (APS). Equipes da Estratégia Saúde da Família (ESF) e da APS, residentes do Município do Rio de Janeiro, Brasil, 2020.

COMPONENTE *	SUBCOMPONENTE	ESTRUTURA	ATIVIDADES	PRODUTOS	RESULTADOS		IMPACTO
					CURTO PRAZO	LONGO PRAZO	
APS	Equipes da ESF e da APS	Recursos humanos **: equipe completa (com número adequado de profissionais de saúde da equipe multiprofissional) e treinada; Recursos materiais: disponibilidade de equipamentos e materiais de consumo em quantidade e qualidade; Recursos físicos: disponibilidade de unidades de saúde em quantitativo adequado para a população adscrita	Realizar consultas de pré-natal; Orientar as gestantes quanto a medidas de prevenção da Zika (uso de repelentes e camisinha) ***; Realizar vigilância sentinela ou o monitoramento de gestantes com suspeita de infecção pelo ZIKV; Realizar e orientar a estimulação precoce das crianças; Realizar exame clínico, neurológico, triagem auditiva, biológica e ocular das crianças; Acompanhar o crescimento e o desenvolvimento das crianças nas consultas de puericultura, atuando na rotina e em possíveis intercorrências; Imunizar as crianças para doenças imunopreveníveis e com vacinas especiais ^#^; Realizar notificação dos casos suspeitos e confirmados de infecção pelo ZIKV em gestantes e de SCZ ao sistema de vigilância em saúde; Realizar a interlocução com as unidades de atenção secundária onde as crianças cadastradas são atendidas em consulta com especialistas e em reabilitação; Integrar as equipes de ESF e do NASF ^##^ com o suporte intersetorial disponível no território: assistência social, coordenação de creches e escolas, terceiro setor (organização não governamental) ^###^ do território, atuando como agente facilitador do acesso a benefícios sociais (renda e transporte), ao acompanhamento pedagógico (creche e escola) e a órteses e próteses	Gestantes com 6 consultas ou mais de pré-natal realizadas; Gestantes e família orientados quanto a medidas de prevenção da Zika (uso de repelentes e camisinha); Gestantes com suspeita de infecção pelo ZIKV monitoradas e acompanhadas; Crianças com estimulação precoce iniciada; Exame clínico, neurológico, triagem auditiva, biológica e ocular das crianças realizados; Crianças em acompanhamento do crescimento e desenvolvimento nas consultas de puericultura; Crianças vacinadas e com calendário vacinal atualizado; Casos suspeitos e confirmados de gestantes com ZIKV e SCZ notificados ao sistema de vigilância em saúde; Agendamento e acompanhamento compartilhado ^§^ entre a APS, as unidades de reabilitação e atendimento especializado realizados; Crianças com alterações no crescimento e desenvolvimento, encaminhadas para a rede de atenção intersetorial	Gestante acolhida, acompanhada e monitorada com qualidade e em tempo oportuno; Redução de gestantes infectadas pelo ZIKV; Estimulação das crianças iniciada em tempo hábil; Diagnóstico precoce de alterações no crescimento e desenvolvimento do feto; Crianças com alterações tardias no crescimento e desenvolvimento detectados precocemente; Crianças imunizadas; Gestantes com infecção pelo ZIKV e crianças com suspeita de SCZ notificadas ao sistema de vigilância em saúde; Crianças com alterações no crescimento e desenvolvimento, com acompanhamento compartilhado ^§^ entre a APS e as unidade de atendimento especializado e em reabilitação; Crianças com alterações no crescimento e desenvolvimento, encaminhadas para a rede de atenção intersetorial com acesso a seguridade social, órteses e próteses	Criança acolhida, acompanhada e monitorada com qualidade e em tempo oportuno	Criança com abordagem precoce das repercussões e melhoria da qualidade de vida

NASF: Núcleo de Apoio à Saúde da Família; ZIKV: vírus Zika.

Nota: elementos do modelo lógico: estrutura, atividade, produto, resultado e impacto. Componentes e subcomponentes: pontos da Rede de Atenção à Saúde (RAS) necessários para o cuidado integral a criança com SCZ; cada elemento, de acordo com os componentes e subcomponentes, tem é formado por itens.

* Os componentes são arranjos da RAS conformados segundo as densidades tecnológicas: APS - nível de menor densidade (*Portaria nº 4.279/2010* do Ministério da Saúde ^74^);

** Recursos humanos na APS: médicos, enfermeiros, dentista, técnicos de enfermagem, técnicos em saúde bucal e agentes comunitários de saúde. Equipe multiprofissional (psicólogo, assistente social, fisioterapeuta, nutricionista, outros);

*** Para a prevenção de novos casos;

^#^ Fornecer acesso a vacinas especiais, disponíveis somente para pessoas de acordo com a patologia e que tenham maior risco de adoecimento;

^##^ Apoio matricial objetiva oferecer retaguarda assistencial especializada e suporte técnico-pedagógico às equipes de referência;

^###^ No Município do Rio de Janeiro: CRAS (Centro de Referência da Assistência Social), CREAS (Centro de Referência especializado da Assistência Social) e CRES (Coordenadoria Regional de Educação), ONGs (organizações não governamentais);

^§^ Projeto terapêutico compartilhado entre as unidades de diferentes níveis de atenção à saúde, de acordo com as necessidades das crianças.

**Quadro 2:** Modelo lógico validado dos componentes da assistência às crianças com síndrome congênita da Zika (SCZ) na atenção primária à saúde (APS). Núcleo de Apoio à Saúde da Família (NASF) e rede de apoio diagnóstico laboratorial, residentes do Município do Rio de Janeiro, Brasil, 2020.

COMPONENTE *	SUBCOMPONENTE	ESTRUTURA	ATIVIDADES	PRODUTOS	RESULTADOS		IMPACTO
					CURTO PRAZO	LONGO PRAZO	
APS	NASF **	Recursos humanos ***: equipe completa (com número adequado de profissionais de saúde da equipe multiprofissional) e treinada para atenção integral à saúde; Recursos materiais: disponibilidade de equipamentos e materiais de consumo em quantidade e qualidade para atenção integral; Recursos físicos: disponibilidade de unidades de saúde em quantitativo adequado para a população adscrita	Realizar a interlocução com as unidades de atenção secundária onde as crianças cadastradas são atendidas em consulta com especialistas e em reabilitação; Integrar as equipes da ESF e do NASF com o suporte intersetorial disponível no território: assistência social, coordenação de creches e escolas, terceiro setor (organização não governamental ^#^ do território, atuando como agente facilitador do acesso a benefícios sociais (renda e transporte), ao acompanhamento pedagógico (creche e escola) e a órteses e próteses; Realizar estimulação precoce das crianças; Realizar consultas interdisciplinares com as equipes da ESF fornecendo apoio matricial **; Realizar acompanhamento dos casos sensíveis à APS e atenção integral à pessoa com doenças crônicas e deficiências	Agendamento e acompanhamento compartilhado ^##^ entre a APS, as unidades de reabilitação e atendimento especializado realizados; Crianças com alterações no crescimento e desenvolvimento, encaminhadas para a rede de atenção intersetorial; Crianças com estimulação precoce iniciada; Crianças acompanhadas em interconsultas pelas equipes do NASF; Acompanhamento dos casos que necessitem do suporte especializado, através de interconsultas com fisioterapeuta, psicólogo, assistente social	Crianças com alterações no crescimento e desenvolvimento, encaminhadas para o atendimento especializado e a reabilitação; Crianças com alterações no crescimento e desenvolvimento, encaminhadas para a rede de atenção intersetorial com acesso a seguridade social, órteses e próteses; Crianças iniciando a estimulação precoce em tempo hábil; Crianças com alterações no crescimento e desenvolvimento detectadas oportunamente	Criança acolhida, acompanhada e monitorada com qualidade e em tempo oportuno	Criança com abordagem precoce das repercussões e melhoria da qualidade de vida
	Rede de apoio diagnóstico laboratorial		Disponibilizar o acesso à rede de diagnóstico laboratorial específico e inespecíficos	Exames realizados	Exames laboratoriais realizados com resultado em tempo hábil		

ESF: Estratégia Saúde da Família.

Nota: elementos do modelo lógico: estrutura, atividade, produto, resultado e impacto. Componentes e subcomponentes: pontos da Rede de Atenção à Saúde (RAS) necessários para o cuidado integral a criança com SCZ; cada elemento, de acordo com os componentes e subcomponentes, tem é formado por itens.

* Os componentes são arranjos da RAS conformados segundo as densidades tecnológicas: APS - nível de menor densidade ( *Portaria nº 4.279/2010* do Ministério da Saúde ^74^);

** Apoio matricial objetiva oferecer retaguarda assistencial especializada e suporte técnico-pedagógico às equipes de referência;

*** Recursos humanos na APS: médicos, enfermeiros, dentista, técnicos de enfermagem, técnicos em saúde bucal e agentes comunitários de saúde. Equipe multiprofissional (psicólogo, assistente social, fisioterapeuta, nutricionista, outros);

^#^ No Município do Rio de Janeiro: CRAS (Centro de Referência da Assistência Social), CREAS (Centro de Referência especializado da Assistência Social) e CRES (Coordenadoria Regional de Educação), ONGs (organizações não governamentais);

^##^ Projeto terapêutico compartilhado entre as unidades de diferentes níveis de atenção à saúde, de acordo com as necessidades das crianças.

**Quadro 3:** Modelo lógico validado dos componentes da assistência às crianças com síndrome congênita da Zika (SCZ) na atenção primária à saúde (APS). Atenção secundária à saúde (ASS), residentes do Município do Rio de Janeiro, Brasil, 2020.

COMPONENTE *	SUBCOMPONENTE	ESTRUTURA	ATIVIDADES	PRODUTOS	RESULTADOS		IMPACTO
					CURTO PRAZO	LONGO PRAZO	
ASS	Atenção especializada	Recursos humanos **: especialistas e equipe multidisciplinar que atuem na reabilitação treinados e em quantitativo adequado para a população de referência; Recursos materiais: disponibilidade de equipamentos e materiais de consumo em quantidade e qualidade para atenção à saúde da criança; Recursos físicos: disponibilidade de unidades de saúde em quantitativo adequado para a população de referência	Realizar consultas com especialistas; Realizar a interlocução com as unidades de atenção primária onde as crianças são cadastradas	Crianças assistidas em especialidades médicas e não médicas; Acompanhamento compartilhado *** entre a APS, as unidades de atendimento especializado realizados	Detecção de alterações tardias, durante o acompanhamento, no crescimento e desenvolvimento; Crianças com alterações no crescimento e desenvolvimento em acompanhamento compartilhado *** com a APS	Criança com SCZ acolhida, acompanhada e monitorada com qualidade e em tempo oportuno	Criança com SCZ com abordagem precoce das repercussões e melhoria da qualidade de vida
	Reabilitação		Disponibilizar vagas para reabilitação; Realizar a interlocução com as unidades de atenção primária onde as crianças são cadastradas	Agendamento em reabilitação realizado; Agendamento e acompanhamento compartilhado *** entre a APS, as unidades de reabilitação	Crianças com reabilitação iniciada precocemente; Crianças com alterações no crescimento e desenvolvimento em acompanhamento compartilhado *** com a APS		
	Rede de apoio diagnóstico de imagem e especializado		Disponibilizar acesso à rede de diagnóstico de imagem (ultrassonografia transfontanela, tomografia computadorizada, ressonância magnética); Disponibilizar acesso à rede de diagnóstico por outros exames: BERA, fundoscopia	Exames realizados	Exames realizados em tempo hábil		

Nota: elementos do modelo lógico: estrutura, atividade, produto, resultado e impacto. Componentes e subcomponentes: pontos da Rede de Atenção à Saúde (RAS) necessários para o cuidado integral a criança com SCZ; cada elemento, de acordo com os componentes e subcomponentes, tem é formado por itens.

* Os componentes são arranjos da RAS conformados segundo as densidades tecnológicas: ASS - nível de densidade tecnológica intermediária (*Portaria nº 4.279/2010* do Ministério da Saúde ^74^);

** Recursos humanos na ASS: disponibilidade de especialistas das áreas médicas: pediatras, neonatologistas, neurologistas, nefrologistas, oftalmologistas, gastroenterologistas, otorrinolaringologistas e ortopedistas. Disponibilidade de especialistas das áreas não médicas: enfermeiros, nutricionistas, fonoaudiólogos, fisioterapeutas, terapeutas ocupacionais, psicólogos e assistentes sociais;

*** Projeto terapêutico compartilhado entre as unidades de diferentes níveis de atenção à saúde, de acordo com as necessidades das crianças.

**Quadro 4:** Modelo lógico validado dos componentes da assistência às crianças com síndrome congênita da Zika (SCZ) na atenção primária à saúde (APS). Atenção hospitalar, residentes do Município do Rio de Janeiro, Brasil, 2020.

COMPONENTE *	SUBCOMPONENTE	ESTRUTURA	ATIVIDADES	PRODUTOS	RESULTADOS		IMPACTO
					CURTO PRAZO	LONGO PRAZO	
Atenção hospitalar	Atenção hospitalar ao parto ** e ao período neonatal	Recursos humanos ***: equipe multidisciplinar que atuem na reabilitação treinados e em quantitativo adequado para a população de referência; Recursos materiais: disponibilidade de equipamentos e materiais de consumo em quantidade e qualidade para atenção à saúde; Recursos físicos: disponibilidade maternidades e de leitos obstétricos e neonatais em quantitativo suficiente para o número estimado de nascimentos na região	Prestar assistência adequada, segundo os protocolos vigentes, ao nascimento e ao período neonatal; Proporcionar suporte básico e avançado de vida de acordo com a necessidade; Realizar exame clínico, neurológico, triagem auditiva, biológica e ocular; Imunizar as crianças para doenças imunopreveníveis e imunobiológicos especiais ^#^; Realizar notificação dos casos suspeitos e confirmados de SCZ ao sistema de vigilância em saúde; Encaminhar as crianças para a unidade de atenção especializada em tempo hábil, quando indicado, em articulação com a APS; Realizar a interlocução com as unidades de atenção primária onde as crianças são cadastradas	Assistência adequada a crianças no nascimento e no período neonatal; Suporte básico e avançado de vida, quando indicado; Exame clínico, neurológico, triagem auditiva, biológica e ocular realizados; Crianças vacinadas; Casos suspeitos e confirmados de crianças com SCZ notificados ao sistema de vigilância em saúde; Crianças com alterações no crescimento e desenvolvimento, com encaminhamento referenciado para as unidades de atendimento especializado e em reabilitação; Crianças com alterações no crescimento e desenvolvimento, com encaminhamento referenciado para as unidades de APS	Atenção adequada ao nascimento e ao período neonatal; Detecção precoce de alterações no crescimento e desenvolvimento; Crianças imunizadas; Crianças com suspeita de SCZ notificadas ao sistema de vigilância em saúde; Crianças com alterações no crescimento e desenvolvimento, encaminhadas para o atendimento especializado e a reabilitação; Crianças encaminhadas para as unidades de APS referenciadas	Criança com SCZ acolhida, acompanhada e monitorada com qualidade e em tempo oportuno	Criança com SCZ com abordagem precoce das repercussões e melhoria da qualidade de vida
	Rede de apoio diagnóstico laboratórial	Recursos humanos: equipe completa e treinada; Recursos materiais: disponibilidade de equipamentos e materiais de consumo em quantidade e qualidade; Recursos físicos: disponibilidade de espaço físico e estruturação adequada	Disponibilizar acesso à rede de diagnóstico laboratorial específico e inespecíficos	Exames realizados	Exames realizados em tempo hábil		
	Rede de apoio diagnóstico de imagem	Disponibilizar acesso à rede de diagnóstico de imagem (ultrassonografia transfontanela, tomografia computadorizada, ressonância magnética); Disponibilizar acesso à rede de diagnóstico por outros exames: BERA, fundoscopia					

Nota: elementos do modelo lógico: estrutura, atividade, produto, resultado e impacto. Componentes e subcomponentes: pontos da Rede de Atenção à Saúde (RAS) necessários para o cuidado integral a criança com SCZ; cada elemento, de acordo com os componentes e subcomponentes, tem é formado por itens.

* Os componentes são arranjos da RAS conformados segundo as densidades tecnológicas: Atenção hospitalar - nível com maior densidade tecnológica (*Portaria nº 4.279/2010* do Ministério da Saúde ^74^);

** De acordo com a estrutura da maternidade, pode ser classificada em atenção secundária ou terciária à saúde;

*** Recursos humanos: organização dos plantões: (disponível 24h/12h/sobreaviso: médicos: obstetra, neonatologista, pediatra, anestesista, generalista, especialista de suporte, enfermeiros: obstetras, neonatologistas, generalistas, técnico de enfermagem, equipe multiprofissional (psicólogo, assistente social, fisioterapeuta, nutricionista, outros);

^#^ Fornecer acesso a vacinas especiais, disponíveis somente para pessoas de acordo com a patologia e que tenham maior risco de adoecimento.

**Quadro 5:** Modelo lógico validado dos componentes da assistência às crianças com síndrome congênita da Zika (SCZ) na atenção primária à saúde (APS). Central municipal de regulação e Vigilância em saúde, residentes do Município do Rio de Janeiro, Brasil, 2020.

COMPONENTE *	SUBCOMPONENTE	ESTRUTURA	ATIVIDADES	PRODUTOS	RESULTADOS		IMPACTO
					CURTO PRAZO	LONGO PRAZO	
Central municipal de regulação	Central de regulação ambulatorial de consultas e procedimentos	Recursos humanos: equipe em quantidade adequada e treinada; Recursos materiais: disponibilidade de equipamentos e materiais em quantidade adequada; Recursos físicos: disponibilidade de espaço físico adequado	Realizar a regulação de vagas de acordo com a classificação de risco	Agendamento de consultas e procedimentos realizados	Crianças com consultas e procedimentos agendados em tempo hábil	Criança com SCZ acolhida, acompanhada e monitorada com qualidade e em tempo oportuno	Criança com SCZ com abordagem precoce das repercussões e melhoria da qualidade de vida
Vigilância em saúde	Núcleos de epidemiologia hospitalar e equipes de vigilância epidemiológica nos níveis local, municipal, estadual e nacional		Realizar a vigilância dos casos de ZIKV por meio de busca ativa de casos, investigação de casos, monitoramento e análise dos casos notificados aos sistemas de informação; Analisar e monitorar os casos notificados nos sistemas de informação; Realizar vigilância ambiental por meio de detecção, bloqueio e monitoramento do índice de infestação do mosquito vetor **; Elaborar e divulgar relatórios sobre a situação de saúde e o acompanhamento dos casos	Relatórios sobre a situação de saúde e o acompanhamento dos casos elaborados e divulgados; Análise e monitoramento dos casos notificados nos sistemas de informação realizados	Monitoramento do perfil epidemiológico dos casos de infecção pela Zika por município, estado, macrorregião e país; Fornecimento de dados epidemiológicos para o planejamento das ações de prevenção, atenção à saúde e vigilância da infecção pelo ZIKV		

ZIKV: vírus Zika.

Nota: elementos do modelo lógico: estrutura, atividade, produto, resultado e impacto. Componentes e subcomponentes: pontos da Rede de Atenção à Saúde (RAS) necessários para o cuidado integral a criança com SCZ; cada elemento, de acordo com os componentes e subcomponentes, tem é formado por itens.

* Os componentes são arranjos da RAS conformados segundo as densidades tecnológicas e setores com ações transversais: Central municipal de regulação e Vigilância em saúde;

** Para a prevenção de novos casos.

Para caracterizar como os componentes do modelo lógico da assistência à saúde da criança com SCZ se articulam, foi elaborada figura que apresenta graficamente o fluxo de gestantes e crianças nos diversos pontos da RAS (Material Suplementar - Figura S5; https://cadernos.ensp.fiocruz.br/static//arquivo/suppl-e00051924_4462.pdf).

## Discussão

Foi elaborado e validado o modelo lógico da assistência integral às crianças com SCZ utilizando metodologia robusta à luz do referencial teórico e análise contextual dos especialistas. Todos os itens foram considerados pertinentes e avaliados como “relevantes” ou “muito relevantes”. Modelos similares não foram identificados na literatura, e o modelo desenvolvido tem o potencial de ser utilizado para o planejamento, monitoramento e avaliação da assistência às crianças com SCZ.

A SCZ é uma doença nova que pode acometer diversos sistemas, levando à necessidade de elaboração de um plano singular de cuidado, demandando visitas frequentes aos serviços de saúde ^1^ ^,^ ^8^ ^,^ ^39^ ^,^ ^49^ ^,^ ^50^. Estudos indicam graves atrasos no desenvolvimento de crianças com SCZ, exigindo assistência básica e especializada ^51^ ^,^ ^52^. Além disso, são apontadas fragilidades para o acesso das crianças em diferentes pontos da RAS, como descontinuidade do cuidado, inexistência de integração e qualificação profissional inadequada, além da ineficiência do sistema de regulação ^53^. Portanto, faz-se necessária a estruturação adequada da RAS, com disponibilidade de unidades de saúde em todos os níveis de complexidade, apoio diagnóstico, protocolos clínicos definidos e fluxos assistenciais adequados.

Apesar dos pressupostos do Sistema Único de Saúde (SUS), são identificadas barreiras no acesso tanto a unidades de saúde quanto a insumos e medicamentos importantes para o tratamento das crianças com SCZ e outras morbidades ^54^. Estudos apontam fragilidades para o acompanhamento de gestantes, dada a cobertura insuficiente de pré-natal, e para o acesso à rede de apoio diagnóstico, bem como para o acompanhamento de crianças com SCZ, vista pela falta de cobertura de atendimento especializado. A organização da RAS, integrada à rede de apoio social, como descrita no modelo lógico, pode contribuir para a mitigação do efeito da SCZ nas famílias, tais como: dificuldade de acesso a serviços de saúde, perda de rendimentos, aumento das despesas e redução da qualidade de vida, além dos gastos pelo Estado com saúde e apoio social ^27^ ^,^ ^50^ ^,^ ^54^ ^,^ ^55^ ^,^ ^56^.

A integração da RAS soma-se a esforços na implementação de políticas para o estabelecimento de direitos, não só das crianças com SCZ como também daquelas com deficiência. Nesse sentido, o plano Viver Sem Limite, iniciativa do Governo Federal, pretende promover ações, como o fornecimento de dispositivos e equipamentos que garantam mais dignidade às pessoas com deficiência ^57^.

A organização, monitoramento e avaliação da rede assistencial é complexa e os modelos lógicos podem auxiliar nessas etapas. Neste estudo, foi elaborado um modelo lógico de fácil entendimento, que objetiva subsidiar a gestão no delineamento e gerenciamento dos fluxos assistenciais direcionados às crianças com SCZ, desde o planejamento estratégico dos recursos e atividades até o monitoramento e avaliação de todas as dimensões do modelo ^18^ ^,^ ^58^.

A maioria dos itens do modelo lógico desenvolvido está relacionada ao componente “Atenção primária à saúde”, demonstrando sua importância como porta de entrada preferencial do SUS, coordenadora do cuidado e ordenadora da RAS ^59^. O cuidado ofertado à criança na APS deve ser iniciado com a assistência pré-natal adequada, orientação às gestantes, diagnóstico da infecção e de possíveis anomalias congênitas e articulação com os demais pontos da rede, especialmente da atenção hospitalar, visando ao parto planejado para melhor assistência ao recém-nato ^39^.

O acompanhamento puericultural oportuno é essencial para o diagnóstico precoce de alterações no desenvolvimento, estimulação precoce e integração com a rede de cuidados e proteção social. No entanto, falhas na coordenação, longitudinalidade, integralidade e orientação da APS já foram apontadas, dificultando a elaboração de um plano de cuidados eficiente e reforçando a necessidade de um modelo lógico para aprimorar a assistência ^27^.

As maternidades devem estar preparadas para acolher gestantes, planejar a via de nascimento e oferecer assistência adequada ao recém-nascido, incluindo insumos para casos graves. É essencial contar com profissionais capacitados para estimulação precoce, cuidados especializados, apoio diagnóstico e alta hospitalar planejada, com encaminhamento para a APS e unidades de reabilitação ^36^ ^,^ ^39^ ^,^ ^60^. Tanto o componente “Atenção primária à saúde” quanto o componente “Atenção hospitalar” mostram a importância da rede de atenção à SCZ estar articulada às redes e políticas de atenção à criança e à mulher. Estudos mostram falhas na assistência pré-natal e ao parto no país, com desfechos perinatais não compatíveis com sua cobertura quase universal, sugerindo problemas na qualidade dessa assistência. A rede de atenção às crianças com SCZ está inserida nessa rede mais ampla de cuidado à saúde da mulher e da criança, sendo o seu funcionamento essencial para o cuidado adequado das crianças com a síndrome.

O componente “Atenção secundária à saúde” foi o terceiro com maior número de itens no modelo, percebendo-se fundamental para o acompanhamento de crianças com SCZ. Sua importância reside na necessidade de um plano de cuidados integrado com a APS, garantindo acesso a exames diagnósticos por imagem, tratamentos especializados, reabilitação e estimulação precoce ^36^ ^,^ ^39^ ^,^ ^61^.

A rede de atenção deve estar estruturada não apenas para a detecção e o tratamento oportuno de crianças com SCZ, mas também para o monitoramento e a identificação precoce de alterações no neurodesenvolvimento em crianças expostas ao ZIKV durante a gestação, mesmo quando assintomáticas ao nascer. O acompanhamento de crianças assintomáticas por dois anos identificou atrasos na cognição, linguagem e desempenho motor ^62^ ^,^ ^63^. No entanto, outro estudo apontou que a maioria das crianças expostas ao ZIKV na gestação apresentava desenvolvimento clínico normal, ressaltando a importância de uma avaliação criteriosa e individualizada ^64^.

O sistema de regulação de vagas deve organizar a demanda de forma equitativa, transparente e com segurança, com classificação de risco para o acesso igualitário das crianças à rede de acordo com suas necessidades em saúde ^65^. O componente “Central municipal de regulação” no modelo é essencial para o fluxo formal de encaminhamento das crianças na rede, embora tenha menos itens. Estudo prévio apontou que a maior parte do apoio prestado às famílias era orientação médica e, embora tenham sido estabelecidas redes de apoio informais, é importante uma rede estruturada, com serviços de saúde e sociais disponíveis para atender às necessidades das crianças ^50^.

O componente “Vigilância em saúde” é transversal a todos os pontos da RAS. Sua atuação mais conhecida é a notificação dos casos de infecção pelo ZIKV e a SCZ, com o monitoramento do perfil epidemiológico ^66^. Entretanto, um sistema de vigilância em saúde deveria incluir o desenvolvimento de uma cadeia de ações para acompanhamento das gestantes e crianças, incluindo a análise da situação de saúde e o adequado funcionamento do sistema de vigilância em saúde com a integração com as equipes de promoção de saúde, prevenção e tratamento ^67^.

Como pontos fortes do nosso estudo, destacamos a extensa revisão da literatura e a incorporação de diferentes áreas de conhecimento; a construção participativa do modelo lógico com profissionais de diferentes áreas de atuação complementares; a validação em duas etapas metodológicas, com avaliação da pertinência e relevância; e a análise estatística através do alfa de Cronbach, que tornaram o modelo mais robusto ^18^ ^,^ ^29^ ^,^ ^30^ ^,^ ^59^ ^,^ ^68^.

A elaboração e validação do modelo lógico consideraram o contexto do Rio de Janeiro, marcado por desigualdades regionais em renda, estrutura urbana e acesso à saúde. Também foram incluídos fatores ambientais e conjunturais que favorecem a propagação do mosquito vetor e a alta incidência de arboviroses ^51^ ^,^ ^69^ ^,^ ^70^ ^,^ ^71^ ^,^ ^72^.

Este estudo teve como principal limitação o baixo percentual (35%) de retorno dos participantes selecionados, o que pode ser decorrente do período de realização - durante a pandemia da COVID-19. Reconhecemos que essa baixa taxa de resposta pode afetar a generalização dos resultados, o que esperamos que tenha sido mitigado pela grande experiência dos profissionais que participaram da validação do modelo em suas atuações nas RAS do SUS e nos temas da avaliação em saúde, da atenção à saúde da criança, e da atenção à pessoa com deficiência, contemplando as áreas de assistência, gestão e pesquisa.

## Conclusão

A construção e validação do modelo lógico dos componentes assistenciais para o cuidado integral às crianças com SCZ no Município do Rio de Janeiro possibilitou a representação gráfica dos componentes necessários para assistência, bem como a organização dos fluxos assistenciais. Esse modelo pode ser utilizado pelos gestores não só para a estruturação da RAS, mas também como uma ferramenta para o monitoramento e a avaliação do cuidado ofertado às crianças com SCZ em todos os níveis da RAS. Ressaltamos que esse é um modelo inicial e que pode ser empregado e adaptado em outros locais com contextos semelhantes, ou seja, de alta densidade demográfica, estruturação urbana precária, recursos físicos e financeiros disponíveis e alta incidência de arbovirose ^73^. Para sua utilização em contextos muito diferentes, seria desejável um novo processo de validação, adaptando o que está expresso na literatura e nas políticas de saúde do país à realidade do território local.
